# Working memory operates over the same representations as attention

**DOI:** 10.1371/journal.pone.0179382

**Published:** 2017-06-12

**Authors:** Ke Chen, Yanyan Ye, Jiushu Xie, Tiansheng Xia, Lei Mo

**Affiliations:** 1 Guangdong Key Laboratory of Mental Health and Cognitive Science, South China Normal University, Guangzhou, China; 2 Center for Studies of Psychological Application, South China Normal University, Guangzhou, China; 3 School of Psychology, South China Normal University, Guangzhou, China; 4 Business School, SunYat-sen University, Guangzhou, China; University of Verona, ITALY

## Abstract

A recent study observed a working memory (WM) Stroop effect with a magnitude equivalent to that of the classic Stroop effect, indicating that WM operates over the same representations as attention. However, more research is needed to examine this proposal. One unanswered question is whether the WM Stroop effect occurs when the WM item and the perceptual task do not have an overlapping response set. We addressed this question in Experiment 1 by conducting an attentional word-color task and a WM word-color task. The results showed that a WM Stroop effect also occurred in that condition, as a word that only indirectly evoked a color representation could interfere with the color judgement in both the attentional task and WM task. In Experiment 2, we used a classic Simon task and a WM Simon task to examine whether holding visuo-spatial information rather than verbal information in WM could interfere with perceptual judgment as well. We observed a WM Simon effect of equivalent magnitude to that of the classic Simon effect. The well-known stimulus-response compatibility effect also existed in the WM domain. The two experiments together demonstrated that WM operates over the same representations as attention, which sheds new light on the hypothesis that working memory is internally directed attention.

## Introduction

Working memory (WM) and attention have been considered to be separate cognitive concepts: WM specializes in the temporary maintenance and manipulation of internal information[1], whereas attention is described as selective focusing on limited information in the environment[2]. Nevertheless, a number of previous studies have found that attention plays an important role in the maintenance and manipulation of WM. For instance, attentional control is a critical faculty that determines WM capacity[3,4]. Other findings have shown that WM can conversely influence visual attention. A representation of a search target held in WM will promote the selection of that target[5]. This phenomenon was explained by the influential biased-competition model[6]. According to this model, representation of the search target maintained in WM will activate related long-term memory representations in the sensory cortex; thus, these neurons will be more easily activated by an external stimulus matching the search target.

Moreover, a growing body of research has shown that even when internally maintained items were task irrelevant, the contents of WM could capture visual attention as well, suggesting that WM and attention mutually influence one another[7–9]. For example, in one study by Soto et al. (2005), participants were asked to remember a colored shape. Subsequently, a search array consisting of several colored shapes with lines embedded in them was presented on the screen. Participants were required to identify a tilted line in one of the colored shapes. The results showed that performance was faster in “valid” trials (i.e., when the memory item appeared in a search array containing the target) and slower in “invalid” trials (i.e., when the memory item reappeared containing a distractor) than in neutral trials (i.e., when the memory item was absent from the search array). Together, eye movement analyses have also shown that first fixation is more likely to land on the target location in valid trials than in invalid trials. This finding indicated that there could be automatic top-down directing of attention to stimuli matching the contents of WM[9]. Such occurrences are still within the scope of explanation of the biased-competition model[7,10]. We can infer from this model that a task-irrelevant WM item can also capture attention just by the same mechanism as a search target kept in WM.

While abundant evidence has shown attentional capture by WM, in some studies, WM content did not necessarily bias attention[11–13]. Whether the linkage between WM and attention is obligatory is controversial. Olivers, Peters, Houtkamp, & Roelfsema (2011) explained why, in some cases, WM content has no or a weak impact on visual search. They proposed that representations in WM are assigned to different activation statuses: active WM (i.e., the representation is within the focus of executive processes) and accessory WM (i.e., the representation is not at the center of attention but still active and accessible)[14]. Olivers et al., (2011) believed that guidance of selection attention could only be induced by active WM presentation but not accessory WM representation. For example, participants must actively keep the search target template in WM when it varied from trial to trial; thus, the other WM representations would be in the accessory status. In such an occasion, task-irrelevant WM content would have a weak or null influence on visual search[12,13].

Recent studied provided more direct supportive evidence for the intimate linkage between WM and attention by showing that classic effects of attention could be replicated in the WM domain. For example, Johnson et al. (2013) observed that “inhibition-of-return” effects in visual attention were also found in the WM domain[15]. Furthermore, short-term memory maintenance also induced a visual aftereffect similar to sensory adaption from prolonged viewing of a visual stimulus[16]. Similarly, Shen et al. (2015) investigated the Ponzo illusion in visual WM[17]. In their study, separate components of a Ponzo figure that were encoded in visual WM sequentially were involuntarily integrated, leading to distorted length perception of the two same-length horizontal lines. In addition to behavioral studies, cognitive neuroscience studies have proposed that WM might have the same neural mechanism as attention. One study found that a search target induced an enhanced N2pc event-related potential (ERP) component when the target-matched item was maintained in WM[18]. The N2pc component has been found to reflect the focusing of attention[19]. In addition, functional magnetic resonance imaging study has shown that stimulus matching WM content enhanced the activity of the superior frontal gyrus, midtemporal, and occipital areas[20], which had previously been known to be sensitive to stimulus reappearance.

These findings cannot simply be explained by the biased-competition model. An emerging account proposes that WM is internally directed attention[21–23]. This account can felicitously explain both the replication of attentional effects in WM and attentional capture by WM. In the study by Kiyonaga and Egner (2014), participants remembered a word written in black ink (either “red,” “green,” “blue,” or “yellow”). After a 2000-ms interstimulus interval (ISI), a rectangular patch was presented on the screen in one of four colors (either red, green, blue, or yellow), and participants were required to report the rectangle’s color. Finally, a WM probe test was presented in which participants answered whether the probe word (either “red,” “green,” “blue,” or “yellow”) matched the WM item. The results showed that participants reported the color of the rectangular patch faster in congruent trials (i.e., when the color of the patch and WM item agreed) than in incongruent trials (i.e., when the color of the patch and WM item differed). This phenomenon was called the WM Stroop effect[24]. Moreover, the congruency effect was of an equivalent magnitude for the classic Stroop task and the WM Stroop task. This finding suggests that maintaining an item in WM could interfere with perceptual judgment in the same manner as that item attended to in the environment, which illustrated that WM operates over the same representations as attention and provided evidence for the hypothesis that WM is internally directed attention.

The study above used a classic Stroop task and WM Stroop task to assess how task-irrelevant information influenced a perceptual task, which is an innovative way to examine the equivalence between WM and attention. The results of that study provided crucial evidence for the proposal that WM operates over the same representations as attention. However, more research is needed to illustrate this momentous hypothesis. The first question we would like to investigate is whether the WM Stroop effect reported by Kiyonage & Egner (2014) could be generalized to a situation where the WM content and intervening color judgment task do not have an overlapping response set. In Kiyonage & Egner (2014), the interference effect in the WM Stroop task could be contributed to by both the stimulus-level and response-level conflict because there was not only sematic overlap between the color-word and ink color but also overlap between the cued manual responses. In Experiment 1, we further investigated whether the WM Stroop effect would occur when there was only sematic overlap but no response overlap between the WM item and the intervening color judgment. We conducted a Stroop-like paradigm that replaced the color-words in Kiyonage & Egner (2014) with non-color-words that have strong color connotations. The results showed that the WM content interfered with the intervening color judgment to the same degree as in the attentional task, even though the stimulus held in WM had no overlapping response set with the color judgment task.

In Experiment 2, we took the investigation a step further to determine whether the visuo-spatial information (rather than verbal information) maintained in WM could also interfere with the perceptual task. Both Kiyonage & Egner (2014) and Experiment 1 in this study used verbal information to investigated whether verbal information encoded in WM could interfere with a perceptual task; hence, whether an interference effect would occur when visuo-spatial information rather than verbal information was maintained in WM is still unanswered. This question was derived from the classic theory of WM, which proposed that verbal information and visuo-spatial information have separate storage mechanisms in WM[1]. Consistent with this model, numerous behavioral and neuroimaging studies have provided evidence for the dissociation of verbal WM and visuo-spatial WM. For example, Smith et al. (1996) found a laterality difference between verbal WM and spatial WM. Spatial WM predominantly involves right-hemisphere regions, whereas verbal WM involves mainly left-hemisphere regions[25]. According to those findings, examining whether the visuo-spatial information held in WM could also have an impact on a perceptual task is necessary and could refine the proposal of the shared representations between WM and attention. In Experiment 2, we adopted the well-known stimulus-response compatibility task, the Simon task, to address this question. We conducted a classic Simon task and a WM Simon task. We observed a WM Simon effect with a congruency effect equivalent to that of the classic Simon effect. The results demonstrated that a stimulus-response compatibility effect also exists in the WM domain.

## Experiment 1

Experiment 1 was conducted to examine whether a WM Stroop effect would occur when there was no overlapping response set between the WM item and the intervening color judgment. We adopted the word-color interference paradigm used in Heurley, Brouillet, Chesnoy, and Brouillet (2013), which required participants to identify the color of a target after being primed with a word that has a color connotation. We modified this paradigm to separate the attentional word-color task from the WM word-color task. Participants completed both tasks in a counterbalanced order.

### Materials and methods

#### Participants

The participants included 28 students from South China Normal University (9 males and 19 females). All participants were native Chinese speakers with a normal or corrected-to-normal visual acuity, and all had normal color vision. The mean age of the participants was 19.65 years with a standard deviation of 2.38 years. Participants received financial compensation after the experiment. This study was approved by the Human Research Ethics Committee of South China Normal University. Informed consent was obtained from participants before the experiment. Participants were also told that they could choose to leave if the experiment made them uncomfortable and that they were free to withdraw from the study at any time.

#### Material and apparatus

E-prime1.1 (Psychology Software Tools, Pittsburgh, PA) was used to program the experimental procedures and collect experimental data. Materials included four Chinese words and two color patches. The stimuli were presented on an IBM ThinkVision C170 16-inch monitor with a spatial resolution of 1024 × 768 pixels at an 85-Hz refresh rate. Participants sat in a quiet and dark laboratory room, sitting approximately 65 cm from the monitor.

Four Chinese words were chosen from a pilot test. In the pilot test, we selected 50 Chinese words in advance, and 50 participants who did not participate in Experiment 1 evaluated the degree to which the words and colors matched (scale from 1 to 7 points, 1 stands for “not at all” and 7 stands for “to a great extent”). The results showed that words well matching the color red were “草莓” (strawberry) and “血液” (blood), with mean ratings over 6. Words well matching the color green were “生菜” (lettuce) and “草坪” (grass), with mean ratings also over 6. These 4 words were chosen as stimuli and represented on the screen in 44-point size font and in Imitation Song-Dynasty-style typeface. The other type of stimulus was the color patch, including a red patch (RGB: 255,0,0) and green patch (RGB: 0,255,0), with a size of 110 mm × 40 mm. All stimuli were presented on a white background.

#### Procedure

All participants completed both an attentional word-color task and a WM word-color task in a counterbalanced order.

**Fig 1:**
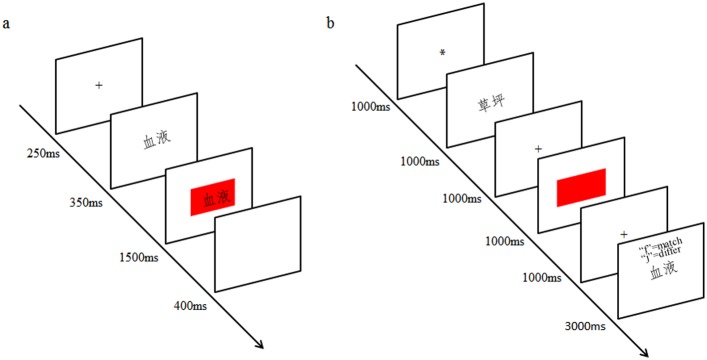
The procedures of the attentional word-color task (a) and the working memory (WM) word-color task (b). Fig 1(a) shows a congruent trial of the attentional word-color task, in which the color related with the word “血液” (blood) was consistent with the red patch. Fig 1(b) shows an incongruent trial of the WM word-color task. The color related with the word “草坪” (grass) and the red color of the patch differed. In the WM probe test, the probe word “血液” (blood) differed from the WM item “草坪” (grass).

*Attentional word-color task*. As shown in Fig 1(a), each trial started with a fixation cross for 250 ms, which was then replaced by one of the four Chinese words for 350 ms. Next, a color patch (red or green) was presented as the background of the word. Participants were required to report the color of the patch as quickly as possible. Half of the participants were required to report “red” by pressing the “f” key and “green” by pressing the “j” key on the keyboard. The response keys were counterbalanced between subjects. The color patch and word disappeared when participants made a response or after 1500 ms. Subsequently, a new trial started after a 400-ms buffer blank. Congruent trials (i.e., the color related with the word was the same as the color of the patch) and incongruent trials (i.e., the color related with the word was different from the color of the patch) occurred in a randomized order. Half of the trials were congruent trials, and the other trials were incongruent trials. Participants completed 8 practice trials with feedback and then finished the following 96 experimental trials without feedback.

*WM word-color task*. As illustrated in Fig 1(b), the WM word-color task contained a color discrimination task and a WM probe test. Each trial began with a fixation point, which was an asterisk, to represent the beginning of a new trial. Then, a word (either “草莓,” “血液,” “生菜,” or “草坪”) was displayed on the screen for 1000 ms (according to Irwin and Yeomans, 1986, a 1000-ms ISI is long enough to ensure that sensory persistence was no longer available[26]), and participants were instructed to keep the word in their memory until this trial finished. After a 1000-ms ISI, a color patch (red or green) was displayed on the center of the screen. Participants were required to report the color of the patch by using the “f” or “j”. The color patch disappeared once participants made a response or after 1000 ms. After a fixation cross was presented for 1000 ms, a WM probe test was presented to test whether participants had actively kept the word in WM or were just shown it. In the WM probe test, one of the four words was displayed on the screen, and participants were instructed to judge whether or not the probe word matched the word maintained in WM. Half of the trials were congruent trials (i.e., the color related with the maintained word was the same as the color of the patch), and other trials were incongruent trials (i.e., the color related with the maintained word was different from the color of the patch). Probe words that matched the WM item and probe words that did not match the WM item also occurred equally often. All trials appeared in a randomized order. Participants completed 12 practice trials with feedback and then finished the following 96 experimental trials without feedback.

#### Results

We conducted a 2 (task: attentional word-color task vs. WM word-color task) × 2 (congruency: congruent vs. incongruent) repeated measures MANOVA to test the impact of conflicting information on the color-discrimination task. The dependent variables were the mean reaction time (RT) and accuracy on experimental trials. The mean RT and accuracy in Experiment 1 are reported in Table 1.

**Table 1:** Mean response time (RT) and accuracy in Experiment 1.

Trial type	Attentional word-color task		WM word-color task	
	RT(ms)	Accuracy (%)	RT(ms)	Accuracy(%)
Congruent	410.65 (51.01)	97.18 (2.71)	513.05 (80.39)	95.31 (4.15)
Incongruent	425.15 (53.22)	97.00 (3.37)	525.29 (73.03)	95.39 (4.83)

*Note*. Standard deviations are given in parentheses. WM = working memory

In the attentional word-color task, the mean RT was included when participants made the correct color judgment, whereas in the WM word-color task, the RT was included only when the WM probe test was also correctly reported. The RT results showed a main effect of task: performance in the attentional color discrimination task was significantly faster than in the WM color discrimination task, *F* (1,27) = 72.297, *p* < .001, *η*^*2*^_*p*_ = .728. The main effect of congruency was also significant, *F* (1,27) = 12.915, *p* < .005, *η*^*2*^_*p*_ = .325. Participants made slower responses in incongruent trials than in congruent trials. There was no interaction between task and congruency. More importantly, the WM word-color task showed a congruency effect of equivalent magnitude as the attentional word-color task (14.5 ms vs. 12.2 ms).

The ACC results showed that the main effect of task was significant, *F*(1,27) = 5.595, *p* < .05, *η*^*2*^_*p*_ = .172. Participants were significantly more accurate in the attentional task than in the WM task. No other main effect or interaction effect was found. Moreover, based on the two-tailed *t*-test on performance on the WM probe test, we found no significant differences between congruent trials and incongruent trials whether performance was measured by mean RT or by accuracy (all *p*s>.1).

Experiment 1 showed that words maintained in WM interfered with the color judgment task. In other words, when the color related with the WM item was different from the color of the patch in the following judgment task, performance was significantly slowed. This result was in accordance with the findings from Kiyonage & Egner (2014). Furthermore, the results of Experiment 1 demonstrated that the WM Stroop effect would occur when an item held in WM has a semantic overlap but no response overlap with the intervening color judgment task. In this experiment, participants kept a word (i.e., strawberry) that only indirectly evoked a color representation and did not overlap with the response in the color judgment task also interfered with the task. Moreover, we observed an interference effect of an equivalent magnitude in the attentional and WM word-color tasks, which implies that an item kept in WM impacted color discrimination in the same manner as if it was in the external environment. The results of Experiment 1 support the hypothesis that WM operates over the same representations as attention. In Experiment 2, we investigated whether visuo-spatial information rather than verbal information (as in Kiyonage & Egner, 2014 and Experiment 1 in this study) held in WM can also interfere with a perceptual task to the same degree as an attentional task. We adopted the classic stimulus-response compatibility paradigm, the Simon task, to examine whether the Simon effect also exists in the WM domain.

## Experiment 2

The classic Simon effect refers to when subjects identify the color of a stimulus faster when the irrelevant spatial location of the stimulus is on the same side as the response key. This is the most well-known stimulus-response compatibility effect. In Experiment 2, we modified the Simon paradigm (cf. Simon & Rudell, 1967) to distinguish between an attentional Simon task and a WM Simon task. Participants completed both of the tasks in a counterbalanced order. We aimed to investigate whether the Simon effect also exists in the WM domain, which could support the idea of shared representations between WM and attention.

### Materials and methods

#### Participants

The participants included 28 students from South China Normal University (9 males and 19 females). The mean age of the participants was 19.76 years, with a standard deviation of 1.18 years. The same requirements in Experiment 1 were used here.

#### Materials and apparatus

E-prime1.1 (Psychology Software Tools, Pittsburgh, PA) was used to program experimental procedures and collect experimental data. Materials were 25.4 mm × 25.4 mm color squares, including a red square (RGB: 255, 0, 0) and a green square (RGB: 0, 255, 0). Stimuli were presented on a black background. An IBM ThinkVision C170 16-inch monitor with a spatial resolution of 1024 × 768 pixels was used to present the stimuli, and the refresh rate was 85-Hz. Participants completed tasks in a quiet and dark laboratory room, sitting approximately 65 cm from the monitor.

#### Procedure

All participants completed both an attentional Simon task and a WM Simon task in a counterbalanced order.

**Fig 2:**
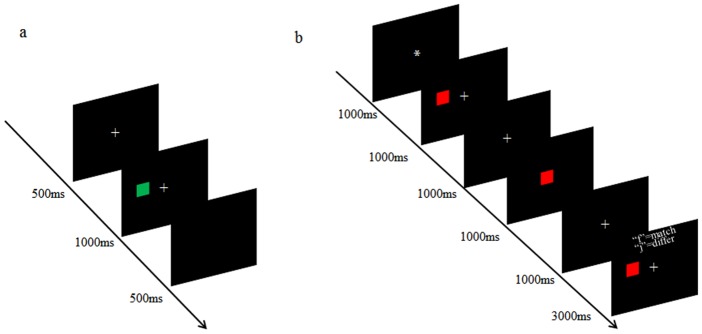
The procedures of the attentional Simon task (a) and the working memory (WM) Simon task (b). Fig 2(a) illustrates an incongruent trial of the attentional Simon task under the condition that participants were required to report the green square by “j” and report the red square by “f”. Fig 2(b) is a congruent trial of the WM Simon task. In this trial, participants were required to press “f” to report the red color of the patch as well as the WM probe that appeared at the same location as the peripheral square.

*Attentional Simon task*. In the classic Simon task, when the stimulus called for a left-hand response, the response was faster when the stimulus appeared on the left-hand side than on the right-hand side[27]. As shown in Fig 2(a), a color square (red or green) appeared on the left or right of the screen (approximately 8.4 cm away from central point). Participants were required to report the color of the color square, regardless of its position. Half of the participants were required to report “red” by pressing “f” using the left index finger (refers to “left”) and “green” by pressing “j” using the right index finger (refers to “right”), and other participants were designated to use the opposite response rule. Half of the trials were congruent trials (i.e., when the location of the color square and the response location were on the same side) and other trials were incongruent trials (i.e., when the location of the color square and the response location were on different sides). Congruent trials and incongruent trials occurred in a randomized order. Participants completed 8 practice trials during which they received feedback and then the following 96 experimental trials without feedback.

*WM Simon task*. The WM Simon task procedure was similar to that of the WM word-color task in Experiment 1, except that participants kept spatial location information in WM rather than a word. As illustrated in Fig 2(b), a color square (red or green) was randomly presented on the left or right of the screen; this was called the peripheral square. Participants kept the location (left or right) of the peripheral square in WM regardless of its color. During the WM delay-period, an intervening task required participants to report the color of a square, which was presented on the center of the screen and had the same color as the peripheral square. Half of the participants reported red by pressing “f” using the left index finger (refers to “left”) and green by pressing “j” using the right index finger (refers to “right”), and the others had the opposite response pattern. In case the color of the center square became predictable for participants, we added filler trials in which the center square had a different color from the peripheral square, accounting for 50 percent of all experiments trials. At the end of the trial, the WM probe test was presented. Participants were asked to judge whether or not the probe square was at the same location as the peripheral square. All other methods were the same as those for the WM word-color task in Experiment 1.

In Experiment 2, we used colored squares rather than other stimuli as a WM cue in order to make the WM Simon task similar to the attentional Simon task. Using other stimuli as the WM cue could possibly create unexpected interference in the WM Simon task compared to that in the attentional Simon task, which would potentially make examining the equivalence of WM and attention a little unfair.

#### Results

We conducted a 2 (task: attentional Simon task vs. WM Simon task) × 2 (congruency: congruent vs. incongruent) repeated measures MANOVA to test whether the Simon effect occurred in the WM domain. The performance measures were the mean RT and accuracy of the experimental trials. The mean RT and accuracy in Experiment 2 are reported in Table 2.

**Table 2:** Mean reaction time (RT) and accuracy for all conditions of Experiment 2.

Trial type	Attentional Simon task		WM Simon task	
	RT(ms)	Accuracy(%)	RT(ms)	Accuracy(%)
Congruent	452.71 (68.74)	97.25 (3.55)	577.93 (67.76)	93.08 (5.62)
Incongruent	466.57 (64.91)	97.57 (2.74)	593.27 (66.35)	91.53 (6.30)

*Note*. Standard deviations are given in parentheses. WM = working memory

Mean RTs in the attentional Simon task were included when participants made a correct response; for the WM Simon task, mean RTs were included only when the WM probe was also correctly reported. We observed a significant main effect of task, *F* (1,27) = 72.672, *p* < .001, *η*^*2*^*p* = .729, and the RT in the attentional task was significantly faster than in the WM task. The main effect of congruency was also significant, *F* (1,27) = 10.790, *p* < .005, *η*^*2*^*p* = .286. For both tasks, participants made faster judgments in congruent trials than in incongruent trials. There was no interaction between task and congruency. Furthermore, the results implied that the attentional Simon effect and WM Simon effect were of an equivalent magnitude (13.85 ms vs. 15.4 ms).

The ACC results showed that the main effect of the task was significant, *F* (1, 27) = 20.71, *p* < .001, *η*^*2*^*p* = .434. Participants were significantly more accurate in the attentional task than in the WM task. There was no other significant main effect or interaction effect. In addition, we found no significant differences in performance on the WM probe test based on a two-tailed *t*-test (all *p*s > .1).

These results indicated that the visuo-spatial information kept in WM interfered with the subsequent perceptual task. Color judgment was slowed when the location information kept in WM differed from the location of the response key. Moreover, we found that there was no difference in the congruency effect between the attentional Simon task and WM Simon task. This result demonstrated a WM Simon effect, which illustrated that the stimulus-response compatibility effect also exists in the WM domain. The location-action link that drove the classic Simon effect also drove the WM Simon effect when the location information was held in WM. The results of Experiment 2 demonstrated that the hypothesis of the equivalence of WM representations and attentional representations is supported in visuo-spatial information.

## Discussion

We conducted two experiments to examine the hypothesis that WM operates over the same representations as attention. In Experiment 1, we found equivalent congruency effects in both an attentional word-color task and WM word-color task, which implied that the WM Stroop effect reported by Kiyonage & Egner could be generalized to a condition in which the remembered item has no overlapping response set with the intervening color judgment task. In Experiment 2, we observed a WM Simon effect of the same magnitude as the classic Simon effect, which implied that holding visuo-spatial information in WM rather than verbal information could also interfere with perceptual judgment. The two experiments together demonstrated the hypothesis that WM operates over the same representations as attention and shed new light on the notion that WM is internally directed attention.

Although numerous former studies have agreed on the relationship between WM and attention, the correlation of WM with attention had not been fully explained. A large number of studies combined a WM and visual search paradigm and observed attentional capture by WM content, which demonstrated the intimate linkage between WM and attention. Kiyonage & Egner (2014) used paradigms to assess the degree of interference that task-irrelevant content exerts on the perceptual discrimination task, which made it possible to assess the equivalence of WM and attention. Their study provided a strong test of the hypothesis that WM and attention operate over the same representations. Based on the study by Kiyonage & Egner (2014), we further examined whether the WM Stroop effect exists when there is only semantic overlap but no response overlap between WM content and color judgment and whether the Simon effect exists in the WM domain when subjects hold task-irrelevant visuo-spatial information in WM. We conducted an attentional task and WM task in both experiments. The WM tasks in both experiments were similar to the attentional task, except for differences in the timing of the conceptual task presentation. In the attentional task, participants made a color judgment while task-irrelevant information was simultaneously visible, whereas in the WM task, participants made a color judgment while task-irrelevant information was no longer present in the environment. We used this method to assess the equivalence of WM representations and attentional representations.

The theory that WM operates over the same representations as attention has been supported by many previous studies[21,28–30]. Based on these studies, the sensory-recruitment theory was proposed, which holds that WM comprises sustained recruitment of stimulus representations in early sensory cortical areas[31,32]. Serences et al. (2009) found sustained activation in the primary visual cortex (V1) during a WM delay period by using functional magnetic resonance imaging[32]. The pattern of activation in the delay period was similar to that of sensory stimuli discrimination. WM representations in V1 were suggested to be “copies” of representations evoked during the sensory process. The results of our research demonstrated the shared representations between WM and attention, which is consistent with the sensory-recruitment theory.

There is also a seemingly divergent finding. In the study by Oliver, Meijer, and Theeuwes (2006), participants first remembered a colored disk and then searched for a diamond-shaped target[8]. The results showed that distractors matching the remembered color induced greater interference than distractors unrelated with WM content in the visual memory condition (i.e., when participants kept the colored disks in visual WM) but not in the verbal memory condition (i.e., when information was kept in verbal WM). WM seemed to only capture visual attention when the memory content was difficult to verbalize. Nonetheless, our findings demonstrated that the proposal of shared representations between WM and attention was supported in both verbal information and visuo-spatial information, which does not fully coincide with the results from Oliver et al. (2006). In their study, the search target would never match the WM item; therefore, participants could strategically avoid distractors matching the WM content. That was probably why there was no greater interference caused by the distractors matching the WM content in the verbal memory condition. In the visual memory condition, the WM test was much more difficult (e.g., distinguish red from a slightly less saturated red and a slightly rustier red) than the verbal memory condition (e.g., distinguish red from blue and green). Participants would draw more resources to maintain the WM item; distractors matching the WM content thus captured more attention in the search task than distractors that did not. Different results in the two memory conditions could be derived from the different strategies.

Although this research was built on the study by Kiyonaga and Egner (2014), our WM probe result did not fully support their result. In the Kiyonaga and Egner (2014) study, a slower response to the WM probe was found in the incongruent trials than in the congruent trials. A time-based resource sharing model (TBRS) was adopted to explain such a phenomenon. This model holds that WM storage and processing rely on the same resource[33]. According to the TBRS model, more attentional resources were needed when making a color judgement in incongruent trials; thus, WM storage would consequently be damaged because of the lack of attentional resources. The congruency effect in the WM Stroop test was as large as 83 ms in Kiyonaga and Egner (2014), implying that there was great conflict in incongruent trials and that more attentional resources were needed for making a color judgment, thus leading to poor WM storage according to the TBRS model. In this study, however, we did not observe a congruency effect of the WM probe test in either Experiment 1 or Experiment 2. This may be due to the small congruent difference in both the WM word-color task and WM Simon task (12.2 ms, 15.4 ms), and WM representations could be refreshed in the 1000-ms-ISI before the WM probe test. Thus, no difference was observed in the WM test between congruent trials and incongruent trials. However, WM probe performance was not what we focused on as it was not related to the “same representation” account. The WM probe results do not undermine the conclusion that WM and attention operate over the same representations.

## Conclusion

To summarize, this study used a word-color interference paradigm and a Simon paradigm to examine the equivalence of WM and attention in both verbal information and visuo-spatial information. We observed a WM Stroop effect in conditions where WM content had no overlapping response set with the intervening perceptual task in Experiment 1 and a WM Simon effect when subjects held task-irrelevant visuo-spatial information in WM in Experiment 2. The two experiments together demonstrated that the hypothesis that WM operates over the same representations as attention is supported in both verbal information and visuo-spatial information.

## Supporting information

S1 File Experiment data of Experiment 1. (RAR)

S2 File Experiment data of Experiment 2. (RAR)
